# Evaluation of a Four Week Interdisciplinary Multimodal Pain Therapy on Chronic Pain Patients—A Comprehensive Approach

**DOI:** 10.3390/life15040576

**Published:** 2025-04-01

**Authors:** Henrike Maria Paulokat, Annett Klinder, Wolfram Mittelmeier, Jörn Bajorat, Katrin Osmanski-Zenk

**Affiliations:** 1 Orthopedic Clinic and Policlinic, Rostock University Medical Center, D-18057 Rostock, Germany; henrike.paulokat@med.uni-rostock.de (H.M.P.); annett.klinder@med.uni-rostock.de (A.K.); wolfram.mittelmeier@med.uni-rostock.de (W.M.); 2 Department of Anesthesiology, Intensive Care Medicine and Pain Therapy, Rostock University Medical Center, D-18057 Rostock, Germany; joern.bajorat@med.uni-rostock.de

**Keywords:** chronic pain, outpatient pain clinic, interdisciplinary multimodal pain therapy, HUBER^®^ 360

## Abstract

Chronic pain syndrome poses a significant challenge to healthcare systems worldwide, affecting millions of individuals and resulting in reduced quality of life and substantial socio-economic costs. This prospective, non-interventional, single-center study evaluated the effectiveness of a four-week interdisciplinary multimodal pain therapy (IMPT) program incorporating neuromuscular training with the HUBER^®^ 360 evolution device. The study included 66 patients diagnosed with chronic pain disorder, divided into an intervention group (*n* = 49) that completed weekly supervised HUBER^®^ 360 sessions and a control group (*n* = 17). Measurements were taken at four time points: day of admission (t1), during therapy (t2), at discharge (t3), and three months post-discharge (t4). The study assessed changes in psychological factors, pain intensity, postural control, and quality of life. The results show that this four-week therapy led to significant improvements in psychological factors such as depression, anxiety, and stress, and significantly reduced the subjects’ pain. The intervention group showed more pronounced improvements compared to the control group. These findings highlight the potential benefits of IMPT in managing chronic pain and improving both physical and psychological health outcomes. This study contributes to the ongoing development of chronic pain management strategies, emphasizing the importance of a multidisciplinary, patient-centered approach. Future research should explore the scalability of IMPT, stratify results based on demographic factors, and evaluate the long-term efficacy of adjunctive tools like the HUBER^®^ 360 device.

## 1. Introduction

Chronic pain syndrome is a major challenge for the healthcare system and affects millions of people worldwide, resulting in reduced quality of life and high socio-economic costs. Effective treatment requires a comprehensive understanding of the underlying mechanisms and interdisciplinary multimodal pain treatment (IMPT).

Pain is a primal sensory perception that acts as a warning signal for the body and indicates an acute injury or trauma. Furthermore, pain signals that a potentially damaging course is imminent but can still be averted [1]. Pain contributes to the preservation of life. Acute pain occurs immediately after an injury or inflammation, acting as a warning signal and contributing to physical integrity. Pain varies greatly between individuals, complicating objective measurement [2]. Acute pain is linked to autonomic and endocrine responses and plays a role in the “fight or flight” response [3,4,5]. It has a clear trigger and persists until the injury or trauma is treated properly and heals [6]. At worst, the pain can change the physiological functions of the body and determine the everyday life and experience of those affected, so that feeling, thinking, and acting are determined by the pain. This point in time describes the onset of chronic pain as a separate diagnosis [5]. It differs from acute pain, as it persists without tissue injury and is considered chronic if it lasts more than three to six months [3,7]. Chronic pain cannot be reduced to a single cause but is usually multi-causal. It affects individuals physically, psychologically, and socially, and is recognized as a disease [3]. It is divided into seven categories, including chronic primary pain, cancer-associated pain, and neuropathic pain [8]. Chronic pain is characterized by increasing duration, less variable pain intensity, and greater spread of pain. It also leads to an elevated use of pain medication, a growing number of visits to the doctor and treatments, and increased restrictions in everyday life and social activities [9].

Risk factors include sociodemographic, psychological, and biological factors [7,10]. Sociodemographic factors include gender, age, weight, smoking, and socioeconomic status. Women are more likely to develop chronic pain and have lower pain thresholds and tolerances, possibly due to hormonal differences like estrogen [7]. Studies have shown that chronic pain prevalence increases with age, particularly in people over the age of 65, making pain management crucial in older populations [10]. Chronic pain is influenced by and can influence mental health, with anxiety, depression, and catastrophizing playing significant roles. Those with other chronic conditions are more prone to chronic pain and related psychological stress [10]. Severe acute pain, if inadequately treated, is a major risk factor for chronic pain itself, as part of the biological risk factors [10]. Effective pain management aims not only to alleviate pain but also to prevent new pain and aid in prevention.

The biopsychosocial model of pain [11,12] acknowledges that chronic pain is not solely a physiological condition but rather a complex interplay of biological, psychological, and social factors. This model underscores the importance of a multidisciplinary treatment approach that integrates medical, psychological, and rehabilitative interventions to address the diverse influences on pain perception and coping mechanisms [12,13]. Such an integrative therapy (interdisciplinary multimodal pain therapy), can help not only reduce pain intensity but also improve patients’ overall quality of life [14].

Chronic pain is the most common reason for doctor visits [3], with varying prevalence rates between 15% and 33% [15,16,17,18,19], leads to high healthcare costs [3,19], and is often inadequately diagnosed and treated.

Interdisciplinary multimodal pain therapy (IMPT) is a comprehensive therapeutic approach to the treatment of chronic pain that combines various medical disciplines and therapeutic approaches. The therapy requires the cooperation of an interdisciplinary team consisting of specialized physicians, psychologists, physiotherapists, occupational therapists, and social workers, who work together to create and implement an individual therapy plan [20,21]. The main aim of IMPT is not to achieve complete freedom from pain, but to make the pain bearable and improve quality of life. IMPT has proven to be superior to unimodal therapy approaches, both in terms of reducing pain and improving the patient’s mood and behavior [12,13,22]. The therapy includes physical, medical, and psychosocial approaches [23].

Studies have shown that IMPT can significantly reduce pain intensity and that the improvements achieved remain stable in the long term [13,23].

The HUBER^®^ 360, used in this study, is an advanced motorized platform developed to improve neuromuscular control, balance, and mobility [24]. Through its multi-axis design and integrated static and dynamic force sensors, it provides precise real-time biofeedback, enabling targeted and effective training [25,26]. Widely utilized in rehabilitation and interdisciplinary multimodal pain therapy, the HUBER^®^ 360 plays a crucial role in enhancing postural coordination, functional strength, and overall mobility [24,27].

To further improve IMPT, the objective of the study was to evaluate the effects of training on the postural control and coordination of patients with chronic pain syndrome, as well as to assess improvements in their pain symptoms and quality of life. The combination of objective measurements and subjective patient interviews enabled a comprehensive evaluation of the therapy effects. This study was intended to show whether similar results could also be achieved for this group of test subjects, how pain patients can be characterized more precisely, and how other factors such as depression, anxiety, and stress develop during IMPT. We hypothesized that a four-week interdisciplinary multimodal pain therapy program incorporating targeted training with the HUBER^®^ 360 device would result in significant reductions in pain intensity and improvements in quality of life for patients with chronic pain compared to conventional therapy approaches. Additionally, the study examined the persistence of these effects three months post-treatment and explored how psychological factors develop during therapy.

The purpose of the study was to provide new insights into the optimization of therapy programs for chronic pain patients and to offer practical implications for future treatments.

## 2. Materials and Methods

In October 2020, the outpatient pain clinic at the University Medical Center Rostock was opened. From 4 January 2021 to 1 July 2022, 80 patients were treated with IMPT, and their therapy outcomes were observed and documented. A pre-therapy assessment in accordance with OPS 1-910 was conducted a few weeks prior, selecting patients for their stay and therapy. OPS code 1-910 is part of the German OPS classification system (Operationen- und Prozedurenschlüssel) and refers to “Interdisciplinary Pain Diagnostic Procedures”. This procedure is used for patients with chronic pain conditions and involves standardized, interdisciplinary diagnostics that consider somatic, psychological, and psychosocial aspects. It is applied to patients who meet at least three of the following criteria: existing or impending impairment of quality of life and/or work ability, failure of previous unimodal pain therapies or pain-related surgeries, existing medication dependence or misuse, pain-sustaining psychological comorbidities, and severe somatic comorbidities. The diagnostic process is led by a specialist physician with additional qualifications in pain therapy and involves collaboration among at least two disciplines, one of which must be psychiatric, psychosomatic, or psychological/psychotherapeutic. The process includes psychometric tests, physical function assessments, and an interdisciplinary team meeting to develop an individualized treatment plan. The goal of this comprehensive diagnostic approach is to address the complex nature of chronic pain and provide a targeted therapy tailored to the patient’s specific needs.

For this prospective clinical study, patients diagnosed with chronic pain disorder (F45.41) were included in the program for appropriate therapy. This prospective, non-interventional, single-center longitudinal study involved interdisciplinary multimodal pain therapy over a four-week period. The multimodal therapy involved a team of specialists, including doctors, psychologists, pain nurses, physiotherapists, occupational therapists, and social workers, all trained in pain medicine. Each time patients visit the clinic, they see various professionals working together on an individualized treatment plan. The therapy is conducted in small groups of up to eight patients.

This study was conducted, as part of the opening of a new outpatient pain clinic at the University Medical Center Rostock, in 2020. Over the course of 1.5 years, this study investigated the effectiveness of an interdisciplinary multimodal therapy program that includes neuromuscular training with the HUBER^®^ 360 evolution device (Human Body Equilibrium 360, LPG Systems, Valence, France).

The measurement points for the study were defined as follows: prior to admission and on the day of admission (t1), during the multimodal pain therapy (t2), at the time of discharge (t3), and 3 months after discharge (t4).

Power analysis was performed on the basis of paired t-test for pain intensity at the beginning and end of the study. As there were no results to refer to, we undertook preliminary study in 6 patients. Analysis of these patients showed the requirement for 66 patients (power = 0.95, effect size = 0.657, *p*-value = 0.05).

### 2.1. Study Cohort

**Figure 1:**
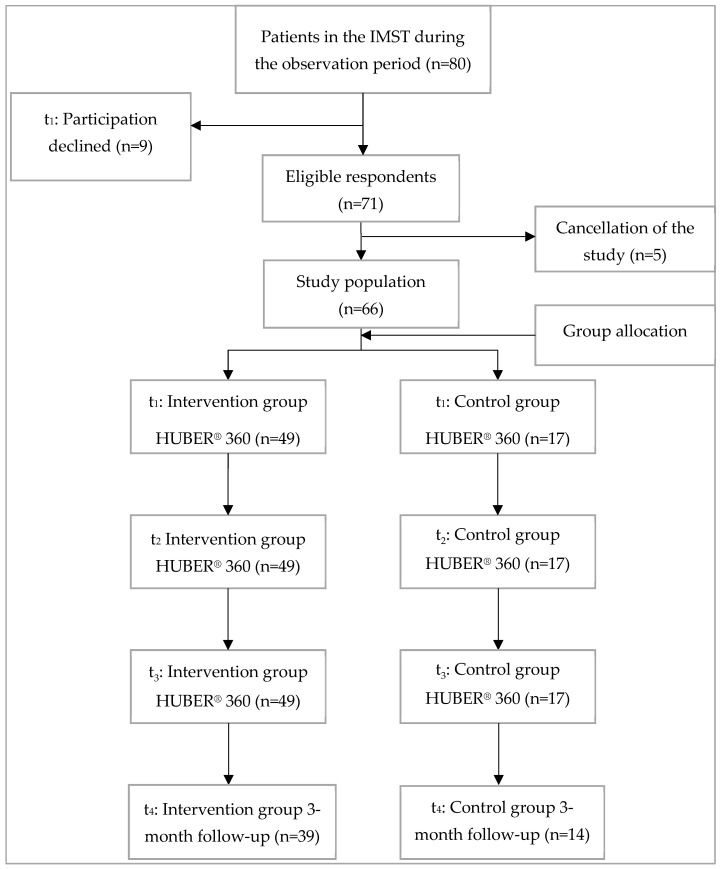
Participant recruitment, t1: before admission and admission day with initial assessment on the HUBER^®^ 360 for both groups; t2: during interdisciplinary multimodal pain therapy (intervention group 3× training on the HUBER^®^ 360; control group no training) t3: discharge with final assessment on the HUBER^®^ 360 for both groups; t4: 3 months after discharge.

This study was a prospective cohort study, where patients with chronic pain were observed over time to evaluate the effects of interdisciplinary multimodal pain therapy. By following patients from admission through therapy and up to 3 months post-discharge, this design allowed for the assessment of both short- and medium-term outcomes. To ensure methodological rigor and transparency, the study adhered to the Strengthening the Reporting of Observational Studies in Epidemiology (STROBE) guidelines [28]. Of the 80 patients in the study period, 66 were included in the study; 14 patients either chose not to participate, had to leave the clinic early due to illness, or were unable to complete the therapy. The 66 patients were divided into two groups based on whether they completed the required weekly training on the HUBER^®^ 360. The intervention group consisted of 49 patients (74.2%) who completed the supervised sessions on the HUBER^®^ 360 Evolution weekly for 4 weeks. The assignment to the groups was based on an interdisciplinary decision made by a team of qualified IMPT staff, considering each patient’s willingness and ability to participate, as well as their health status and mobility. Patients deemed suitable for regular HUBER^®^ 360 training were assigned to the Intervention group, while those for whom regular participation was not feasible formed the Control group. This classification reflects real-world therapy adherence rather than random assignment. Training protocols emphasized dynamic postural exercises tailored to individual needs, leveraging real-time feedback from the device to optimize neuromuscular engagement. The exercises progressed in complexity, addressing the heterogeneity of chronic pain presentations and fostering adaptive neuroplasticity. The control group consisted of 17 patients (25.8%), who completed fewer than three sessions. All 66 patients underwent initial and final evaluations on the HUBER^®^ 360 for comparative analysis (Figure 1).

### 2.2. Inclusion and Exclusion Criteria

The diagnosis F45.41 (chronic pain disorder with somatic and psychological factors), classified under the ICD-10 by the World Health Organization (WHO) [8], was an inclusion criterion. It describes persistent pain lasting at least 6 months, influenced by both physical and psychological factors, without a fully explained medical cause. This condition significantly impacts daily life, work, and social roles, reducing quality of life. Patients had to be sufficiently independent and physically and cognitively able to participate in the coordination test and willing to partake in the follow-up. To enhance generalizability, the sample encompassed diverse demographic characteristics, including age, gender, and comorbidities.

Exclusion criteria referred to patients with conditions requiring other specific diagnostics or treatments, those unavailable for follow-up, and patients with severe obesity (body-mass index (BMI) ≥ 40).

### 2.3. Ethics and Data Privacy

An ethics application was submitted on 10 November 2020, with approval granted on 24 November 2020 (A 2020-0269). Patients were informed verbally and in writing about the study, its background, and objectives, and provided consent for data collection and participation. They could withdraw from the study at any time without giving reasons.

### 2.4. Statistical Analysis

Data were pseudonymized in Microsoft Excel and analyzed using SPSS 29.0 (IBM, Armonk, NY, USA). Descriptive analysis included mean, standard deviation, minimum, maximum, and count for quantitative variables, and absolute and relative frequencies for qualitative variables. Specific information about the used statistical test is provided with the respective results. Briefly, pretreatment patient data between the treatment and control groups were either analyzed with unpaired *t* test (normally distributed data) or with Mann–Whitney U test (not normally distributed data), with significance set at *p* < 0.05. Normal distribution was assessed with the Shapiro–Wilk test. Chi-^2^ tests compared distributions of qualitative parameters between groups.

Comparison of patient-relevant outcomes was performed in SPSS using a general linear model with repeated measures, with time points and HUBER^®^ 360 treatment as the relevant factors (two-way ANOVA). Subsequent post hoc test with Bonferroni correction for the single main effects indicated changes between time points or between the groups. The *p* values for the “time x treatment (HUBER) effect” as well as significant changes in the post hoc test were reported.

Graphical visualization of NRS data was created with GraphPad Prism 9.0 (GraphPad Software, Boston, MA, USA), including results from the comparison of the different time points per group as well as the comparison of the different groups at each time point. The analysis in GraphPad Prism 9.0 was performed as mixed model (two-way ANOVA), which allowed missing values for the repeated measures of the individuals, including Bonferroni post hoc tests.

### 2.5. HUBER^®^ 360

The HUBER^®^ 360 is a multi-axis, motorized training platform used for neuromuscular analysis, training programs, and rehabilitation. Manufactured by LPG SYSTEMS, the multi-axis 360-degree platform is patented by the LPG group, France. The acronym HUBER stands for Human Body EquilibRium. The HUBER^®^ 360 can detect the position, center of gravity, and force of users through built-in static and dynamic force sensors in the platform and handles [26]. These measurements are visualized in real time to provide direct biofeedback during training [25]. The device was used to train in the following categories: flexibility and mobility, dynamic strengthening, posture and balance, and endurance [26]. During the four-week therapy at the outpatient pain clinic, initial and final evaluations were conducted on the HUBER^®^ 360, with three intermediate training sessions. Training sessions lasted between 25 and 40 min, and the evaluations took about 15 min each. All participants, regardless of their underlying condition, age, or gender, performed the same exercises on the HUBER^®^ 360 to achieve better comparability among subjects. The only difference was between the control and intervention groups, with the intervention group completing weekly training sessions.

### 2.6. Outcome Measures

Increasingly, it is important to measure treatment success not only through objective parameters but also by focusing on the subjective treatment quality perceived by the patients. Therefore, Patient Reported Outcome Measures (PROMs) are used to measure and compare subjective health status, as in this study.

### 2.7. Questionnaires

1. German Pain Questionnaire (DSF) and Depression, Anxiety, and Stress Scale (DASS): The DSF is widely used in pain therapy settings to collect baseline data and develop tailored therapy concepts. It includes demographic data (age, gender, weight, height), subjective pain descriptions (occurrence, frequency, intensity, location), questions about daily life, medical history, psychological factors (depression, stress, anxiety, quality of life), and social data (education, insurance, employment, family, retirement) [29,30]. In this study, DSF version 2015.2, which includes the DASS, was used. Comorbidities were recorded and the Charlson Comorbidity Index calculated [31]. To assess the development of pain intensity and the success of therapy for patients in the outpatient pain clinic and beyond, various follow-up questionnaires of the DSF were used [32], such as the progress questionnaire, which was sent to the patients 3 months after intervention.
2. Numerical Rating Scale (NRS): The NRS asks patients to rate their pain on an 11-point scale from 0 (no pain) to 10 (maximum pain) [33]. During the therapy, pain intensity was recorded every morning using the NRS for a total of 20 days, to tailor the therapy and evaluate its success.
3. Mainz Pain Staging System (MPSS) by Gerbershagen: The MPSS is a coding system used to stage chronic pain based on temporal and spatial aspects, medication behavior, and patient history [34,35]. This classification facilitates the comparison of chronic pain patients and the provision of appropriate therapy. The MPSS is widely used in Germany, as it allows quick and simple evaluation from patients’ data, showing a strong correlation between pain chronification, psychological condition, and daily life impairment. High chronic pain stages often correlate with significant work disability [36].
4. Graded Chronic Pain Status (GCPS) by von Korff: The GCPS is an internationally accepted method for assessing chronic pain based on pain intensity and disability. The classification by von Korff uses pain intensity, disability, persistence, and frequency to categorize chronic pain in a straightforward manner [37].
5. European Quality of Life 5 Dimensions 3 Level Version (EQ-5D-3L): Developed by the EuroQol Group, this questionnaire assesses health-related quality of life across five dimensions: mobility, self-care, usual activities, pain/discomfort, and anxiety/depression [38]. An extra question about current health status was added for this study. A visual analog scale (EQ-VAS) is included, where patients rate their health on a scale from 0 (worst imaginable health) to 100 (best imaginable health).
6. Short-Form 36 Health Survey (SF-36): The SF-36 is widely used to measure health-related quality of life (HRQOL) across eight subcategories, with an additional question on health changes over the past year. Subcategories include physical functioning, physical role, emotional role, social functioning, bodily pain, vitality, mental health, and general health perception. It includes two summary scores: physical health component (PCS) and mental health component (MCS) [39,40,41]. Higher scores indicate less impairment and better quality of life. Chronic pain significantly impacts these scores, particularly with increased pain severity, leading to lower HRQOL [41,42].

## 3. Results

### 3.1. Descriptive Data

The sample consisted of 52 (78.8%) women and 14 (21.2%) men. They were on averaged 56.6 ± 13.2 years old (Table 1). The patients were asked about their current occupation, with less than half (42.4%) stating that they were employed. Around half (*n* = 32; 48.5%) of the respondents were currently drawing a pension. The respondents receiving a pension were asked whether their retirement was temporary or permanent. Here, 21 (65.6%) stated that they were already permanently retired. However, not all of them were receiving a pension due to reaching the age limit.

More than half (56.1%) of the respondents answered yes to the question about an existing disability (Table 2). Of these, around 54% had a degree of disability of 50 or more.

Each week, patients participated in a psychotherapy session, three physiotherapy sessions, six occupational therapy sessions, one session on the HUBER^®^ 360 device, and two consultations with their doctor. Additionally, they had access to individualized sessions such as Tai Chi, nutritional counseling, biofeedback, and stress management.

Comparison between the intervention and control groups, using either the chi-squared test or Fisher’s exact test, showed no statistically significant differences in any of the parameters presented in Table 2. This indicated that the groups were comparable, thus minimizing potential confounding factors that could influence the study results. The Charlson Comorbidity Index (Table 1 and Table 2) is used to calculate the 10-year survival rate of patients. Accordingly, patients with a CCI of 0 have a 10-year survival rate of 98.3%. In the intervention group, about 60% (*n* = 29) of the patients had a 10-year survival rate of more than 90%, and only about 10% (*n* = 5) had a rate of less than 10%. In the control group, on the other hand, around 50% (*n* = 8) of the participants had a 10-year survival rate of more than 90%, and around 30% (*n* = 5) had a survival rate for the next 10 years of less than 10%.

### 3.2. Pain

The pain intensity of participants was measured at two points (t1 and t3) using DSF data. Scores between 0 and 49 indicated low pain, while those between 50 and 100 indicated high pain. Both groups started with high pain levels. After four weeks of therapy, the intervention group’s average pain intensity decreased from 71.47 to 51.5, while the control group’s average dropped slightly from 72.51 to 55.75. However, the two-way ANOVA showed no statistically significant difference (*p* = 0.556).

A two-way ANOVA revealed a significant improvement (*p* = 0.002) in the intervention group between day 1 and the last day of therapy (day 20) according to NRS. In the control group, a significant improvement (*p* = 0.01) was only observed between days 5 and 20.

Both groups started with similar pain levels, with a gradual decrease over time. In the intervention group, the mean NRS was 5.71 ± 1.94 on day 1, dropping to 4.24 ± 1.77 on day 20. The control group started with a slightly higher mean NRS of 5.76 ± 1.79 on day 1, which decreased to 4.47 ± 1.7 by the end of the therapy. Overall, pain intensity decreased over the four weeks, with a more pronounced reduction in the intervention group.

**Figure 2:**
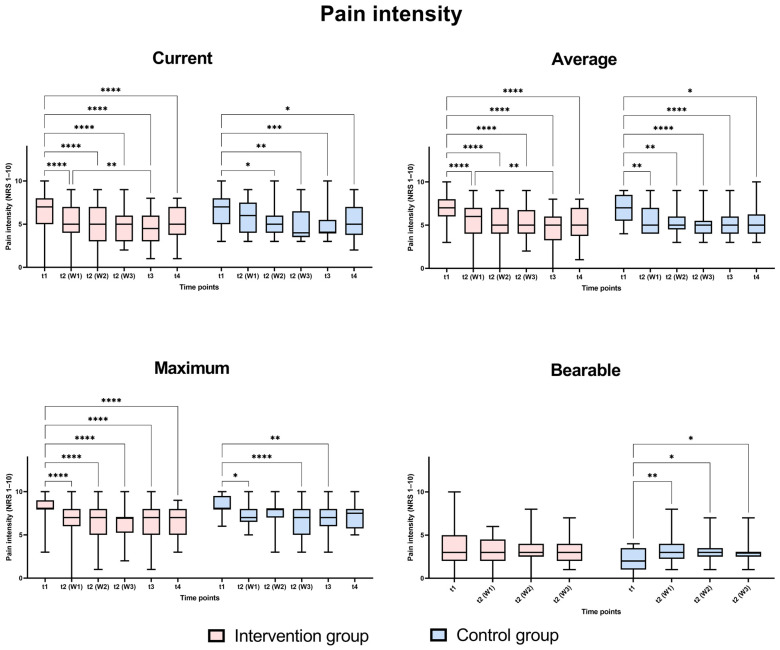
Comparison of pain levels at different time points; t1 (day of admission), t2 (W1): week 1, t2 (W2): week 2, t2 (W3): week 3, t3 (at discharge), t4 (three months post-discharge). Analysis was performed as mixed model two-way ANOVA with Bonferroni post hoc tests, *p*-values: *: *p* < 0.05, **: *p* < 0.01, ***: *p* < 0.001, ****: *p* < 0.0001.

The different pain levels were analyzed in the intervention and control groups over time using mixed analysis two-way ANOVA for related samples to identify significant changes in the pain levels at different points as well as between the groups at each time point. Figure 2 shows that there was a significant change in pain intensity. These changes are reflected particularly strongly in the direct comparison before therapy (t1) and at the end of therapy (t3). The effect observed three months post-therapy, while still present, was less pronounced compared to the initial impact observed at the beginning of the intervention. There were no significant differences in the assessed pain levels between the Huber training group and the group without extra training at any of the analyzed time points.

### 3.3. Patient Satisfaction and Quality of Life

#### EQ-5D-3L

Quality of life measured using the EQ-5D-3L (Table 3) improved on average during IMPT. The intervention group showed significant improvements from the start to the end of therapy. Three months after the end of therapy, this value fell slightly, but did not return to the initial value at the start of therapy. The same was also recorded for the EQ-5D-3L visual analog scale.

### 3.4. Psychological Parameters

#### 3.4.1. Short Form-36

The Short Form-36 health questionnaire (Table 4) was completed twice by all patients. The mean value for both groups for the physical component summary increased and showed significant improvements. A significant improvement over time was also measured for the mental component summary. An overall significant improvement was also documented in the SF-36 total score in both groups. With the help of the SF-36 questionnaire, an increase in the physical and psychological perception of the pain patients could be registered.

#### 3.4.2. Depression Anxiety Stress Score (DASS)

A significant improvement in depression, anxiety, and stress (Table 5) was recorded for the intervention group across all three categories. Significant improvements in the categories of depression and stress were also recorded for the control group.

## 4. Discussion

This study enabled a detailed characterization of chronic pain patients diagnosed with F45.41 and provided insights into the potential benefits of interdisciplinary multimodal pain therapy (IMPT). Furthermore, it offered an opportunity to refine and optimize the established IMPT concept. Although the sample size of 66 patients is small, the data align with similar studies from Germany and Europe [13,17,43,44], offering valuable insights into the characteristics, behaviors, and treatment options for chronic pain patients.

Our study, in line with others on chronic pain patients [10,17,45,46], found that women are diagnosed with chronic pain more frequently than men. Women accounted for 78.8% of participants, compared to 21.2% men. This aligns with previous research [10,46,47] suggesting that biological, psychological, and social factors—including hormonal differences, pain processing, and societal roles—may contribute to this disparity. Notably, female patients exhibit higher pain sensitivity, influenced by hormones like estrogen and testosterone [10]. In our study, women were more likely to initiate participation in IMPT, potentially due to their more frequent healthcare visits. Some male participants reported being encouraged by their partners to enroll. Importantly, no significant gender differences were observed between the intervention and control groups, ensuring unbiased comparison of therapy outcomes. In the discussion of gender differences in chronic pain prevalence and treatment, it is essential to consider not only biological factors but also the social and psychological dimensions of the biopsychosocial model [11]. While research suggests that women generally have lower pain thresholds and higher pain sensitivity [46], this explanation alone does not fully account for their higher representation in interdisciplinary pain clinics. Social and cultural factors play a critical role in shaping how pain is perceived, expressed, and managed in clinical settings [11]. Studies indicate that gender stereotypes influence pain reporting and treatment-seeking behavior [48]. Women are often more likely to express pain and seek medical attention, whereas men may underreport symptoms due to societal expectations of emotional restraint and independence [49]. Gender stereotypes like ’men need to be physically strong’ [49] can result in men’s pain being underestimated in medical settings, reinforcing gender disparities in healthcare [49]. These cultural stereotypes may contribute to the underrepresentation of men in interdisciplinary pain clinics, rather than an actual lower prevalence of chronic pain. Additionally, healthcare providers may have implicit biases that affect referral patterns, potentially leading to disparities in access to specialized pain management services. To present a more comprehensive and balanced perspective, it is important to acknowledge these factors and consider why men may be less represented in pain clinics. Future research should explore how gendered socialization and healthcare practices influence pain treatment and access to interdisciplinary pain therapy. By addressing these biases, pain management strategies can be improved to ensure equitable care for all individuals, regardless of gender.

Chronic pain patients undergoing IMPT span a wide age range, from young adults to the elderly. Age-specific factors influence the causes and management of chronic pain. For older adults, degenerative conditions such as osteoarthritis, osteoporosis, and disc degeneration are common contributors, often coupled with psychological challenges like depression or anxiety stemming from isolation or loss. In younger adults, chronic pain may result from injuries, accidents, or inflammatory diseases [17]. While chronic pain is more prevalent among older adults, our participants ranged from 27 to 82 years, showing that chronic pain is not exclusive to the elderly. The control group had an average age of 61.8 years, compared to 54.7 years in the intervention group. Although this age difference may reflect reduced postural control and motivation for certain therapies, it was not statistically significant (*p* = 0.058), allowing for meaningful comparisons between groups. Lifestyle factors significantly influence chronic pain, with evidence showing that physical inactivity, stress, poor sleep, and unhealthy diets exacerbate pain through neurophysiological mechanisms and mood deterioration [10]. Todd et al. [16] highlighted that chronic pain is more prevalent among individuals with lower socioeconomic status, limited access to healthcare, and higher exposure to psychosocial stressors. Our study confirmed similar trends, as participants exhibited high stress levels, poor sleep quality, psychosocial burden, and reduced physical activity, all of which are known to contribute to pain chronification.

Integrating lifestyle interventions such as physical activity, stress management, improved sleep hygiene, and anti-inflammatory diets into pain management can enhance outcomes and improve patient quality of life [10].

The study confirmed that patients with chronic pain experienced a reduction in pain intensity during their participation in the IMPT program. The average pain intensity on the Numeric Rating Scale (NRS) decreased from 5.73 to 4.3, with significant improvement in the intervention group (*p* = 0.002). Daily NRS assessments allowed for better insights into pain development and potential influencing factors. While pain levels increased slightly after therapy, they remained below pre-therapy levels. The therapy provided tools to help patients manage pain beyond the clinical setting [44]. Chronic pain was predominantly multifactorial [5,11,13], with common causes including specific illnesses, surgeries, and physical or psychological stressors. Early detection of pain chronification, especially post-surgery or illness, is crucial to prevent progression. Most participants were in advanced stages of chronic pain (Stage 3 according to the “Mainzer Stadienmodell der Schmerzchronifizierung”) [50] or classified as Severity Grade 4 (von Korff) [9], aligning with findings from other studies. Pain was most commonly reported in the lower back, hips, knees, and lower extremities [44]. This emphasizes the need for therapy tailored to the pain’s location, as patients with lower-extremity pain encountered more challenges using the HUBER^®^ 360 system. Chronic pain duration was long-lasting, with over half of participants suffering for more than five years [17], underscoring the importance of early intervention to prevent worsening conditions.

At the start of therapy, psychological well-being was assessed using the FW7 questionnaire, revealing significant mental health challenges in both groups. Depression emerged as a prominent comorbidity of chronic pain, emphasizing the need for early monitoring and treatment to prevent its reciprocal influence on pain [14]. The SF-36 questionnaire [40] tracked physical and mental health over four weeks. Both groups showed significant improvements in physical (Intervention: *p* < 0.001; Control: *p* = 0.003) and mental health scores (both groups: *p* < 0.001). By the end of therapy, participants approached the mental health benchmarks of a healthy population [42], while physical health, though improved, remained below average. These findings underline the need for multidisciplinary care addressing both physical and psychological aspects to enhance quality of life for chronic pain patients. Data from the DASS showed significant reductions in depression (*p* < 0.001), stress (*p* < 0.001), and anxiety levels during therapy. At baseline, 44% of participants exhibited high depression scores, which dropped to 10.7% by the end of therapy. The HUBER^®^ 360 Evolution device had no significant impact on psychological outcomes, suggesting its effects are limited to physical parameters like mobility and strength. Larger studies are needed to explore its potential psychological benefits. Avoidance behavior can worsen pain, whereas past abuse or violence can increase the likelihood of experiencing chronic pain [51]. Chronic pain often leads to depressive symptoms, impacting daily life and work, potentially causing disability or job loss, and leading to social withdrawal. These psychosocial consequences can further exacerbate the condition. Comorbidities can also lead to depression, with about 20% of chronic pain patients experiencing depressive symptoms [18]. The findings highlight the critical role of psychosocial factors, including stress, anxiety, depression, and social isolation, in chronic pain. Evidence from other studies shows that 95% of chronic pain patients initially exhibit psychological comorbidities [44]. IMPT centers are encouraged to integrate psychological support, behavioral therapy, and stress management techniques to address these factors effectively.

During the four-week IMPT program, both groups showed improvements in quality of life (QoL), as measured by the EQ-5D-3L, consistent with findings from a similar Swedish study [43]. Multimodal therapy helped patients enhance their QoL, maintaining or slightly exceeding baseline levels. The intervention group demonstrated significant improvements during therapy, though some deterioration occurred post-therapy. However, their QoL at the three-month follow-up remained higher than at baseline. Daily, close supervision likely contributed to these positive outcomes, suggesting that continued support after therapy could help sustain higher QoL levels. Differences between EQ-5D-3L and SF-36 results in the control group may reflect the instruments’ varied sensitivity and focus [52]. The EQ-5D-3L evaluates five broad dimensions (mobility, self-care, daily activities, pain/discomfort, and anxiety/depression) with only three response levels, making it less sensitive to subtle changes [38]. In contrast, the SF-36 assesses eight detailed dimensions, covering both physical and psychological health [40], enabling a more nuanced analysis, especially in psychosocial aspects like vitality and emotional well-being. Psychological adjustments, such as coping strategies and pain acceptance, may have improved psychosocial dimensions, which were captured more precisely by the SF-36. A potential ceiling effect in the EQ-5D-3L might explain the absence of significant progress in the control group if a stable baseline had already been achieved [52]. Additionally, the intervention group completed three extra HUBER^®^ 360 sessions, likely enhancing physical dimensions of QoL. These differences highlight the complementary nature of the two questionnaires and their varying ability to detect changes. While the SF-36 captured more granular improvements, the EQ-5D-3L provided a broader, less detailed perspective [52]. The findings emphasize the need for tailored interventions and sensitive measurement tools to assess and sustain long-term improvements in QoL.

Few studies have examined the effectiveness of the HUBER^®^ 360 Evolution. However, significant improvements have been noted in neuromuscular control, mobility, balance, and muscle strength. One study presented at the “Congress of Rheumatology of the European League against Rheumatism” found that HUBER^®^ 360 training yielded better pain relief, muscle strengthening, and quality of life improvements in chronic back pain patients compared to conventional rehabilitation [24]. Another study reported significant improvements in trunk flexibility, muscle endurance, and pain reduction in patients with nonspecific chronic back pain after a six-week intensive rehabilitation program with the HUBER^®^ 360 [27]. The findings of this study provide partial support for the statement regarding the effectiveness of the HUBER^®^ 360 Evolution. In the context of IMPT, the HUBER^®^ 360 device was utilized for neuromuscular training, showing improvements in physical parameters such as postural control and coordination. However, its specific impact on broader outcomes like muscle strength, trunk flexibility, or endurance, as mentioned in prior studies, was not directly assessed in this research.

While this study demonstrated significant reductions in pain intensity and improvements in quality of life and psychological factors for the intervention group, the HUBER^®^ 360’s role appeared more complementary within the multimodal approach. No independent assessment of the device’s efficacy compared to conventional rehabilitation methods was conducted, which limits its direct comparability to the cited studies [24,27]. Therefore, while the results align with previous findings [24,27,53] on the HUBER^®^ 360’s potential benefits, particularly for enhancing physical metrics within a structured therapeutic framework, they do not provide definitive evidence to confirm or contradict the specific claims about its superiority in pain relief, muscle strengthening, or quality of life improvement compared to conventional methods. Further targeted studies isolating the device’s effects are necessary for a conclusive evaluation. To draw more definitive conclusions about the HUBER^®^ 360’s unique impact, future research should isolate its effects from other components of IMPT and include direct comparisons with alternative rehabilitation techniques. This would help clarify whether the improvements observed in neuromuscular control and pain relief can be attributed solely to the device or to the broader multimodal therapeutic context.

### Limitations and Strengths

This study has several methodological limitations. The sample size of 66 participants is relatively small compared to similar studies, potentially limiting statistical significance. The broad diagnostic category (F45.41) includes patients with diverse pain profiles, reducing population homogeneity and comparability. Additionally, the classification in this study was based on clinical judgment rather than random assignment, which may introduce some degree of selection bias. Since participation in the training was determined by the interdisciplinary team based on patients’ health status, mobility, and willingness, external factors such as motivation or pre-existing functional differences could have influenced group allocation. However, by ensuring that both groups were comparable in key demographic and clinical characteristics, we aimed to minimize potential biases and maintain the validity of the study results.. Follow-up assessments relied solely on telephone interviews and questionnaires without re-evaluation on the HUBER^®^ 360 device, potentially affecting data accuracy and long-term effect assessment. In addition, conclusions regarding the durability of the observed effects are limited by the single-center design—which may limit the generalizability of the results—and the lack of long-term follow-up.

Despite these limitations, the study offers valuable strengths. Comprehensive data collection enables a detailed characterization of chronic pain patients and insights into pain chronification and therapy effectiveness. Continuous monitoring throughout the study period, rather than only at baseline and post-intervention, allows for precise tracking of patient progress. A three-month follow-up supports the assessment of treatment sustainability and potential relapses.

Despite methodological constraints, this study provides important insights into chronic pain treatment. The findings contribute to understanding therapeutic effects, guiding future research, and optimizing clinical practice. They support evidence-based improvements in pain management and promote personalized therapeutic strategies. The study offers promising perspectives for further research and clinical application.

## 5. Conclusions

This study underscores the significant benefits of interdisciplinary multimodal pain therapy (IMPT) in managing chronic pain, improving both physical and psychological health outcomes for patients. Results revealed notable reductions in pain intensity, depression, anxiety, and stress, coupled with improved postural control and quality of life. While the intervention group exhibited more pronounced improvements, findings highlight the necessity of personalized and sustained therapeutic strategies to maximize long-term benefits.

Future research should explore the scalability of IMPT, stratify results based on demographic factors like age and gender, and evaluate the extended efficacy of adjunctive tools such as the HUBER^®^ 360. Additionally, integrating lifestyle modifications and addressing psychosocial dimensions could further enhance outcomes for chronic pain patients. This study contributes to the ongoing evolution of chronic pain management, emphasizing the importance of a multidisciplinary and patient-centered approach.

## Figures and Tables

**Table 1:** Descriptive data of both groups in comparison; SD: standard deviation, BMI: Body Mass Index, CCI: Charlson Comorbidity Index, NRS: Numerical Rating Scale, *p*-value: significance level,^1^: *t* test, ^2^: Mann-Whitney U test.

	Intervention Group (*n* = 49)		Control Group (*n* = 17)		Group Comparison t1
	Mean (±SD)	Median (Range)	Mean (±SD)	Median (Range)	*p*-Value
Age	54.7 (±12.4)	56 (27; 79)	61.8 (±14.5)	60 (38; 82)	0.058 ^1^
BMI	26.7 (±5.95)	24.49 (17.9; 40)	29.07 (±5.6)	29.2 (18.3; 37.2)	0.107 ^2^
CCI	1.57 (±1.58)	1 (0; 6)	2.47 (±2.15)	2 (0; 6)	0.178 ^2^
NRS day 1	5.71 (±1.94)	6 (1; 9)	5.76 (±1.79)	6 (3; 9)	0.964 ^2^

**Table 2:** Descriptive frequencies of the population.

		Intervention Group (*n* = 49)	Control Group (*n* = 17)
		Absolute (Percentage %)	Absolute (Percentage %)
Sex	female	40 (81.6%)	12 (70.6)
	male	9 (18.4)	5 (29.4)
Charlson Comorbidity Index	0	14 (28.6%)	4 (23.5%)
	1	15 (30.6%)	4 (23.5%)
	2	9 (18.4%)	1 (5.9%)
	3	6 (12.2%)	2 (11.8%)
	4	0 (0%)	1 (5.9%)
	5	4 (8.2%)	4 (23.5%)
	6	1 (2%)	1 (5.9%)
Disability	Yes	27 (55.1%)	10 (58.8%)
	No	22 (44.9%)	7 (41.2%)
Cause of pain (multiple answer options)	no recognizable cause	6 (12.2%)	1 (5.9%)
	specific disease	23 (46.9%)	6 (35.3%)
	operation	16 (32.7%)	9 (52.9%)
	accident	5 (10.2%)	3 (17.6%)
	physical strain *	22 (44.9%)	2 (11.8%)
	mental stress	17 (34.7%)	3 (17.6%)
	other cause	5 (10.2%)	0 (0%)
Duration of pain	1 month to ½ year	2 (4.1%)	1 (5.9%)
	½ year to 1 year	4 (8.2%)	2 (11.8%)
	1 to 2 years	5 (10.2%)	1 (5.9%)
	2 to 5 years	11 (22.4%)	5 (29.4%)
	more than 5 years	27 (55.1%)	8 (47.1%)
Mainz Pain Staging System (MPSS)	stage 1	0 (0%)	0 (0%)
	stage 2	3 (6.1%)	1 (5.9%)
	stage 3	46 (93.9%)	16 (94.1%)
Graded Chronic Pain Status (GCPS) by von Korff	grade 1	1 (2%)	1 (5,9%)
	grade 2	8 (16.3%)	1 (5.9%)
	grade 3	9 (18.4%)	2 (11.8%)
	grade 4	31 (63.3%)	13 (76.5%)

*: *p*-value 0.03; The statistically significant difference in pain due to physical strain was excluded from the main analysis because the study focused on therapy effectiveness, not baseline pain causes. Since both groups received the same treatment, variations in pain etiology were unlikely to impact outcomes.

**Table 3:** EQ-5D-3L of the intervention group and control group compared at time points t1, t3, t4. SD: standard deviation, *p*-value: significance, ^1^: significant change within the intervention group from t1 to t3, ^2^: significant change within the intervention group from t3 to t4.

		Intervention Group			Control Group				2-Way ANOVA *p*-Value (Time X HUBER^®^ 360)
		t1	t3	t4	t1	t3	t4	*p*-Value	
EQ-5D-3L Index Value	mean (±SD)	0.55 (±0.30)	0.78 (±0.18)	0.68 (±0.28)	0.64 (±0.26)	0.73 (±0.22)	0.65 (±0.29)	<0.001 ^1^	0.263
	median (range)	0.564 (0.11; 1)	0.877 (0.175; 1)	0.788 (0.11; 1)	0.701 (0.175; 0.887)	0.788 (0.175; 0.887)	0.788 (0.175; 0.887)	0.036 ^2^	
EQ-5D-3L Visual Analog Scale	mean (±SD)	49.82 (±17.38)	62.14 (±15.1)	55,5 (±19.13)	47.53 (±12.5)	55.12 (±17.76)	54.57 (±18.22)	0.003 ^1^	0.696
	median (range)	50 (10; 100)	60 (30; 100)	55 (4; 95)	48 (25; 70)	60 (15; 85)	55 (9; 75)	0.014 ^2^	

**Table 4:** SF-36 score of the intervention group and control group at time points t1, t3 in comparison. SD = standard deviation, *p* = significance, ^1^: *p*-value between intervention and control group at time point t1, ^2^: *p*-value between intervention and control group at time point t3, ^3^: *p*-value within the intervention group between t1 and t3, ^4^: *p*-value within the control group between t1 and t3.

		Intervention Group		Control Group			Two-Way ANOVA *p*-Value (Time X HUBER^®^ 360)
		t1	t3	t1	t3	*p*-Value	
Physical component summary	mean (±SD)	34.54 (±19.1)	48.65 (±21.28)	28.62 (±11.66)	40.75 (±17.51)	0.235 ^1^ 0.174 ^2^ <0.001 ^3^ 0.003 ^4^	0.668
	median (range)	31.25 (6.25; 85.5)	44.75 (7.5; 89)	28 (15.5; 63)	37 (9.25; 84.75)		
Mental component summary	mean (±SD)	48.32 (±22.72)	58.09 (±23.57)	53.37 (±19.86)	69.96 (±16.49)	0.419 ^1^ 0.060 ^2^ <0.001 ^3^ <0.001 ^4^	0.191
	median (range)	3.25 (3.75; 91.5)	61.55 (3.75; 94.25)	45.17 (21.75; 89.75)	71.38 (40; 97.75)		
SF-36 overall	mean (±SD)	41.43 (±18.58)	53.37 (±20.62)	40.99 (±13.54)	55.36 (±15.43)	0.929 ^1^ 0.718 ^2^ <0.001 ^3^ <0.001 ^4^	0.567
	median (range)	38.23 (7.75; 88.5)	53.88 (5.63; 88.25)	37.88 (19.25; 76.38)	51.38 (27.13; 91.25)		

**Table 5:** Depression, anxiety, stress score of the intervention group and control group at time t1 and t3 in comparison. SD: standard deviation, *p*-value: significance, ^1^: *p*-value between intervention and control group at time t1, ^2^: *p*-value between intervention and control group at time t3, ^3^: *p*-value within the intervention group between t1 and t3, ^4^: *p*-value within the control group between t1 and t3.

		Intervention Group		Control Group			Two-Way ANOVA *p*-Value (Time X HUBER^®^ 360)
		t1	t3	t1	t3	*p*-Value	
Depression	mean (±SD)	9.65 (±5.11)	3.63 (±4.02)	7 (±3.59)	2.12 (±2.52)	0.053 ^1^ 0.151 ^2^ <0.001 ^3^ <0.001 ^4^	0.402
	median (range)	9 (0; 21)	2 (0; 20)	8 (2; 12)	2 (0; 10)		
Anxiety	mean ± (SD)	5.69 (±3.96)	3.14 (±3.27)	3 (±2.94)	2.71 (±3.31)	0.013 ^1^ 0.637 ^2^ <0.001 ^3^ 0.703 ^4^	0.014
	median (range)	4 (0; 16)	2 (0; 15)	3 (0; 11)	2 (0; 12)		
Stress	mean (±SD)	10.8 (±4.86)	5.88 (±4.15)	8.35 (±3.71)	3.71 (±2.31)	0.064 ^1^ 0.045 ^2^ <0.001 ^3^ <0.001 ^4^	0.836
	median (range)	10 (2; 21)	5 (0; 16)	8 (2; 16)	3 (1; 10)		

## Data Availability

The data presented in this study are available upon request from the corresponding author. The data are not publicly available but can be obtained from the Department of Clinical Research in the Orthopedic Department of the University Medicine Rostock if required.
